# High-Risk Lipoprotein(a) Levels in Saudi Women and Its Relationship to Menopause and Adiposity

**DOI:** 10.3390/nu15030693

**Published:** 2023-01-30

**Authors:** Nouf Aljawini, Lateefa O. Aldakhil, Syed Shahid Habib

**Affiliations:** 1 Department of Physiology, College of Medicine, King Saud University, Riyadh 11416, Saudi Arabia; 2 Department of Community Health Sciences, College of Applied Medical Sciences, King Saud University, Riyadh 11433, Saudi Arabia; 3 Department of Obstetrics and Gynecology, King Saud University Medical City, King Saud University, Riyadh 11416, Saudi Arabia

**Keywords:** lipoprotein(a), menopause, dyslipidemia, body adiposity, cardiometabolic risk, Saudi

## Abstract

Lipoprotein(a) is an inherent CVD risk biomarker that varies by race, and the levels of Lp(a) in Saudi women are relatively unexplored. We aimed to examine the effect of age and menopause on Lp(a) and explore the correlation between adiposity and cardiometabolic risk factors with Lp(a) in Saudi women. The third aim was to determine the predictors of elevated Lp(a) in this population. In this cross-sectional study of 229 women, we compared Lp(a) serum levels, adiposity indices, and lipid and glycemic profiles between menopausal groups. We used immunoturbidimetry to measure serum Lp(a) and BIA to assess body composition. We evaluated the relationship between Lp(a) and our parameters using ANOVA and Spearman’s correlations. Regression was used to determine the predictors of high-risk Lp(a) levels. The mean of Lp(a) was 28.37 mg/dL, and the concentration increased significantly in postmenopausal (premenopausal 20.98 ± 12.30; perimenopausal 29.92 ± 9.53; postmenopausal 32.49 ± 9.83 mg/dL; *p* < 0.001. High-risk levels were 57.1% in postmenopausal and 19.1% in premenopausal. The magnitude of Lp(a) increased significantly after age 50. Lp(a) was significantly associated with age and cholesterol and negatively associated with % FFM. Lp(a) increased by 0.41 units for every year of age, indicating the strongest correlation.

## 1. Introduction

Lipoprotein(a) [Lp(a)] is identified as a cardiovascular risk factor independent of traditional risk factors [1,2] and plays a role in prothrombotic and atherogenic processes [1,3]. Lp(a) is a novel risk factor for aortic valve stenosis [1]. Lp(a) is composed of two components: an LDL-like particle and an apolipoprotein(a) [Apo(a)]. The high molecular weight of apo accounts for the differences in size and electrophoretic mobility between Lp(a) and LDL. Lp(a) concentrations are genetically determined primarily by the apo(a) gene and vary significantly between individuals [4].

Genetic and observational studies show a causal relationship between elevated Lp(a) concentration and atherosclerotic cardiovascular disease, aortic valve stenosis, and all-cause mortality in men and women and across ethnic groups [1]. Lp(a) levels and population-level distribution differ significantly by ethnicity [5]. Among all ethnic groups studied, black people have the highest Lp(a) level [5]. Lifestyle accounts for approximately 25% of Lp(a) level variation [6]. Despite the strong genetic influence on Lp(a) levels, some studies have found an increase in Lp(a) levels with age, particularly in women over 50. In contrast, others suggested that women’s Lp(a) levels rise during menopause [7]. The transition to menopause and the years preceding the final menstrual period (FMP) are marked by estrogen fluctuation and, eventually, estrogen deficiency, which has been linked to a proatherogenic lipid profile [8].

Estrogen hormone inhibits LPA gene transcription [9], and elevated Lp(a) induces the expression of inflammatory genes in the vascular cells [1]. Menopause is associated with increases in serum total cholesterol, low-density lipoprotein cholesterol (LDL-C), apolipoproteins, and triglycerides, decreases in high-density lipoprotein cholesterol (HDL-C), and increases in Lp(a) concentrations [8]. It is unclear whether aging or hormonal changes cause this menopause-related increase in Lp(a). However, it should be noted that women on hormone replacement therapy (HRT) have lower Lp(a) levels than women who are not on HRT [7]. Fogacci et al. systematically evaluated the effects of different anti-estrogen therapies on plasma Lp(a) levels in postmenopausal women from 10 clinical trials, including 1128 women in the active-treated arms and 921 women in the control arms. A meta-analysis of the data shows that anti-estrogen therapy for women significantly lowered Lp(a) [10].

An increased risk of metabolic and cardiovascular disease is associated with changes in body composition during menopause [11]. The SWAN study reported that accelerated fat mass gain and loss of fat-free mass were related to the menopause transition [12]. Body composition changes may also vary depending on race or initial adiposity when entering perimenopause. Women gain about 2 to 3 kg during menopause [11,13]. Despite changes in body composition, some women may not gain weight [11]. Dyslipidemia is linked to postmenopausal abdominal fat gain. Within a year of the FMP, women in the SWAN study experienced significant increases in total cholesterol, low-density lipoprotein cholesterol (LDL-C), and apolipoprotein (Apo)B concentrations [14].

The plasma level of Lp(a) in Saudi postmenopausal women is unknown, as is the relationship of plasma Lp(a) level with the menopausal transition. To our knowledge, no previous studies have assessed the relationship between the plasma concentration of Lp(a) and menopausal status in Saudi women. Therefore, our primary aim was to examine the effect of age and menopausal status on Lp(a) levels. We hypothesized that menopausal transition is associated with increased Lp(a) and body adiposity. A secondary aim was to explore the correlation of body adiposity indices, including body mass index (BMI), body fat percentage(%BF) and fat-free mass percentage (%FFM), and cardiometabolic risk factors, including lipid and glycemic profile with Lp(a) levels in premenopausal, perimenopausal, and postmenopausal stages. A tertiary aim was to determine the clinical predictors of elevated Lp(a) in this population.

## 2. Materials and Methods

### 2.1. Study Design and Settings

This was a cross-sectional study reported according to the Strengthening the Reporting of Observational Studies in Epidemiology (STROBE) recommendations [15]. This study was conducted in the Physiology and Obstetrics & Gynecology departments at King Saud University (KSU) and King Saud University Medical City (KSUMC) in Riyadh from March 2022 to December 2022. Ethical approval was obtained from The Institutional Review Board of KSU college of medicine (No. E-21-5998), and all women provided written informed consent. Research was conducted in agreement with Helsinki Declaration guidelines for experiments involving human beings [16]. Anthropometric parameters, metabolic profile, body composition, and lipoprotein(a) levels were compared between premenopausal, perimenopausal, and postmenopausal women.

### 2.2. Participants and Protocol

Women who visited the KSUMC Obstetrics and Gynecology clinics and completed the study procedures were eligible to participate. In total, according to the standard criteria, 229 women were recruited and grouped into premenopausal, perimenopausal, and postmenopausal groups. The premenopausal, perimenopausal, and postmenopausal ages ranged from 30 to 45, 47 to 52, and 54 to 67, respectively. Pregnant women or women with a history of myocardial infarction, stroke, familial hypercholesterolemia, chronic kidney disease, chronic liver diseases, cancers, or autoimmune disorders were excluded.

### 2.3. Menstrual History Measures

We used semi-structured interviews to collect menstrual history information from all women. Menopausal status was evaluated based on bleeding pattern and compared as a categorical variable, with premenopausal, perimenopausal, and postmenopausal groups defined using the Stages of Reproductive Aging Workshop criteria [17]. Women were classified as premenopausal by having regular cycles, perimenopausal by having irregular cycles with an interval of amenorrhea of more than 60 days or more than two skipped cycles, and postmenopausal by the absence of a menstrual cycle for more than 12 consecutive months.

### 2.4. Study Procedures

#### 2.4.1. Anthropometrics Measurements and Body Adiposity Assessment

Anthropometrics and body composition were assessed using the study protocol’s standardized operating procedures. Women’s weights and heights were measured using a Seca electronic scale (Seca 576; SECA CORP, Hamburg, Germany). The body mass index (BMI) was calculated by dividing the weight in kilograms by the square of the height in meters (kg/m^2^) and categorized using the World Health Organization standards (WHO) [18]. Body fat percentage (%BF) was chosen as a surrogate marker for total body adiposity. Tanita bioelectrical impedance analysis (BIA) was used to assess body composition (Tanita MC-980, Tokyo, Japan). We estimated body fat percentage (%BF) by assessing body composition using BIA as its convenience and performance have been validated in previous studies compared to dual X-ray absorptiometry [19].

#### 2.4.2. Plasma Lipoprotein(a) and Lipid Profile Measurement

Fasting blood samples were collected in 3.2% (0.109 mol/L) sodium citrate tubes at enrollment and centrifuged at 1100× *g* for 15 min within 2 h of venous sampling. Each resulting plasma was then divided into ten aliquots of 100 μL plasma and frozen at −80 °C for later analysis. Total serum cholesterol (TC), high-density lipoprotein cholesterol (HDL-C), and triglycerides (TG) were analyzed using enzymatic lipoprotein assays by autoanalyzer (Dade Behring, Inc., Deerfield, IL, USA). The Friedewald equation was used to determine the (LDL-C) levels [20].

Glycated hemoglobin (HbA1c) was analyzed by ion exchange high-performance liquid chromatography (HPLC) using the GX assay for the Tosoh Automated Glycohemoglobin Analyzer (HLC-723GX, Tosoh Co., Tokyo, Japan).

Lipoprotein(a) was analyzed by turbidimetric immunoassay with the kits quantex Lp(a) supplied by Biokit Spain.

The measurement in our investigation was traceable to the International Standardization Reagent SRM2B, which is accepted by the World Health Organization (WHO) Expert Committee on Biological Standardization as the First WHO/International Reference Reagent for Lipoprotein(a) Immunoassay (IFCC) [21]. Lp(a)was measured by a Hitachi 911 machine manufactured by (Roche diagnostics, Indianapolis, IN, USA). For Lp(a), the limit of quantification (LOQ) was 1.3 mg/dL, and the limit of detection (LOD) was 0.4 mg/dL.

### 2.5. Statistical Analysis

Statistical Package for Social Sciences (SPSS) version 20.0 (IBM Corp., Armonk, NY, USA).was used for data entry and analysis. Descriptive characteristics and the biochemical profile were expressed as Mean ± SD (Standard Deviation). Kolmogorov–Smirnova and Shapiro–Wilk tests were used to see whether the data followed a normal distribution or not. Those parameters not following normal distribution (skewed data) were analyzed by the non-parametric Mann–Whitney test for two groups and the Kruskal–Wallis test for more than two groups. For multiple group comparisons, ANOVA (analysis of the variance) was used for normally distributed data. Spearman’s and Pearson’s correlations were determined between different parameters. Stepwise linear regression models were created to determine the independent predictors of Lp(a) levels to see how the variance explained R2 changes by adding (or removing) each predictor. All *p*-values were two-tailed, and those less than 0.05 were considered statistically significant.

## 3. Results

### 3.1. Clinical and Biochemical Characteristics

A total of 229 women enrolled. All of them provided information about their menopausal status. The study included 68 premenopausal, 63 perimenopausal, and 98 postmenopausal women. Table 1 summarizes the study sample’s anthropometric, clinical, and biochemical characteristics. The average age of premenopausal women was 37.41 (SD± 7.22); the average age of perimenopausal women was 49.55 (SD ± 1.63); and the average age of postmenopausal women was 60.45 (SD ± 6.33). In terms of the main variables examined, the mean (standard deviation) of lipoprotein(a) was 28.37 (11.60) mg/dL, and the %BF was 41.21 (6.57).

### 3.2. Body Adiposity Parameters Associated with Menopausal Status

In terms of body mass index (BMI), body fat percentage (%BF), and fat-free mass percentage (%FFM), there are significant differences between menopausal groups. As shown in Table 1, the postmenopausal women had a higher BMI (premenopausal 30.64 ± 4.70; perimenopausal 32.13 ± 5.89; postmenopausal 33.60 ± 6.23 kg/m^2^; *p* = 0.005) and higher %BF (premenopausal 39.01 ± 6.04; perimenopausal 40.95 ± 8.01; postmenopausal 42.91 ± 5.39; *p* = 0.001) than the premenopausal and the perimenopausal group. However, fat-free mass percentage (%FFM) was highest in premenopausal and lowest in postmenopausal women (premenopausal 60.98 ± 6.04; perimenopausal 59.04 ± 8.01; postmenopausal 57.08 ± 5.39; *p* = 0.001). In, terms of fat mass index (FMI), fat-free mass index (FFMI), and normalized fat-free mass index (NFFMI), postmenopausal women had the highest level of FMI, and the difference between the groups was statistically significant. At the same time, there were no statistical differences between the menopausal groups in FFMI and NFFMI.

### 3.3. Lipoprotein(a) Level Associated with Menopausal Status and Age

**Figure 1:**
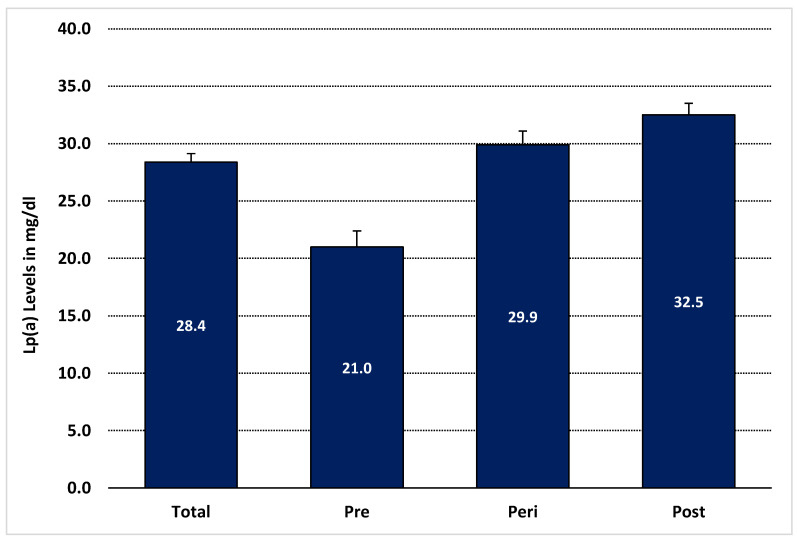
Comparing lipoprotein(a) level in premenopausal, perimenopausal, and postmenopausal women. Note: Data are presented as the mean of lipoprotein(a) level in mg/dL in each group according to menopausal status. Abbreviations: Lp(a): lipoprotein(a); Total: Total sample; Pre: premenopausal women; Peri: perimenopausal women; Post: postmenopausal women.

**Figure 2:**
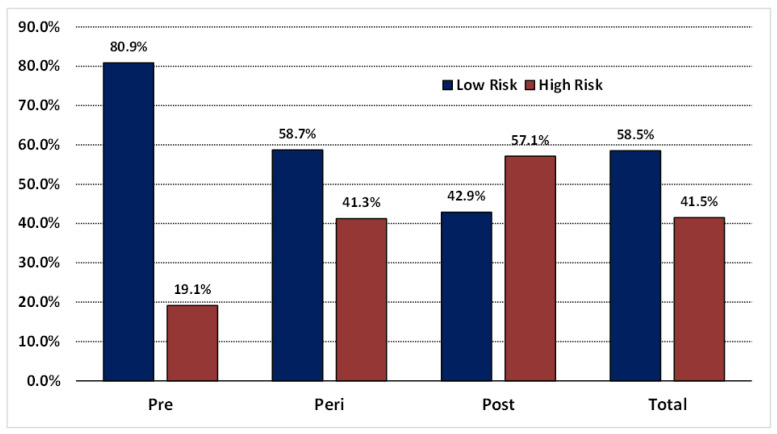
The distribution of low-risk and high-risk lipoprotein(a) levels among premenopausal, perimenopausal, and postmenopausal women; Lp(a) cut-off value is 30 mg/dL. Note: Data are presented as the percentage of low-risk Lp(a) level and high-risk Lp(a) level among menopausal groups. Lipoprotein(a) cut-off value is 30 mg/dL. The low-risk group’s Lp(a) concentration is below 30 mg/dL. The high-risk group’s Lp(a) concentration is above 30 mg/dL. Abbreviations: Pre: premenopausal women; Peri: perimenopausal women; Post: postmenopausal.

The study looked at plasma lipoprotein(a) levels in Saudi women according to menopausal status and age. As shown in Table 1, the average Lp(a) level in the total sample was 28.37 (SD± 11.60) mg/dL. Lp(a) concentration increased significantly postmenopause, as illustrated in Figure 1. There were significant differences between menopausal groups in terms of Lp(a) concentration, with lower Lp(a) in the premenopausal group compared to the perimenopausal and postmenopausal groups (premenopausal 20.98 ± 12.30; perimenopausal 29.92 ± 9.53; postmenopausal 32.49 ± 9.83 mg/dL; *p* < 0.001) Table 1. Furthermore, the study looked at the distribution of low risk of Lp(a) levels (less than 30 mg/dL) and high-risk Lp(a) levels (more than 30 mg/dL) among menopausal groups, as shown in Figure 2. A total of 57.1% of postmenopausal women are in the high-risk score, while 19.1% of premenopausal women are at the high-risk amount of Lp(a) concentration. In our sample, the mean age of premenopausal women was 37.41, and the mean age of postmenopausal women was 60.45 years.

**Figure 3:**
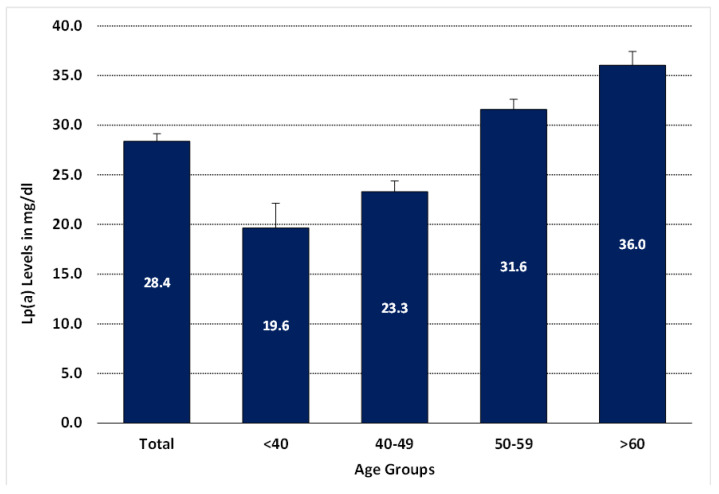
Comparing lipoprotein(a) levels in different age groups. Note: Data are presented as the mean of lipoprotein(a) level in mg/dL in each bar according to age groups.

**Figure 4:**
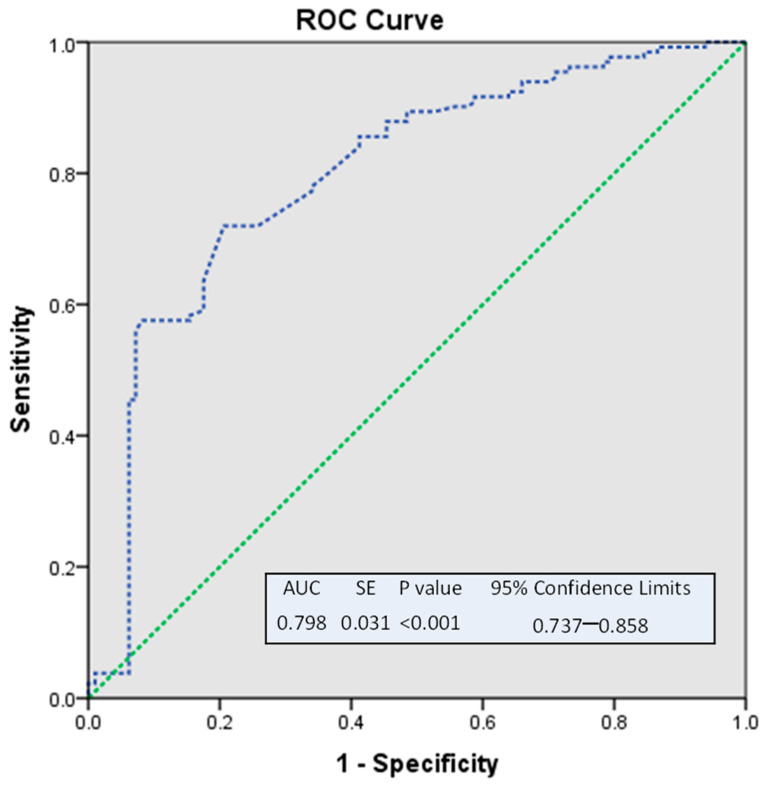
ROC analysis of age as a predictor of lipoprotein(a) levels in all participants. Note: We dichotomized the age into two groups based on the cut-off of 50 years old [7]. ROC analysis was performed to see the predictive value for Lp(a) levels. It revealed AUC 0.798, *p* < 0.001. It showed that age significantly strongly predicts high Lp(a) levels in women. Abbreviations: ROC curve: receiver operating characteristic curve; AUC: area under the receiver operating characteristic curves.

When categorizing Lp(a) level into four age groups, as shown in Figure 3, the magnitude of Lp(a) increased significantly after age 50 and was reported to be the highest in women above 60. In Figure 4, we dichotomized the age into two groups based on a cut-off of 50 years [7], as reported before. ROC analysis was performed to see the predictive value for Lp(a) levels. It revealed AUC 79.8%, *p* < 0.001. It showed that age significantly and strongly predicted elevated Lp(a) levels in women.

### 3.4. Correlations among Lipoprotein(a) Levels and Clinical Risk Factors

The relationship between the lipoprotein(a) level and the clinical risk factors was examined using Spearman’s Rank Correlation in all women, premenopausal, perimenopausal, and postmenopausal groups. Of the nineteen factors examined, eleven significant correlations were observed in Table 2, with the strongest correlations observed between Lp(a) and age (r = 0.488, *p* < 0.01). Lp(a) concentration correlated significantly positively with adiposity indices, BMI (r = 0.137, *p* < 0.05), %BF (r = 0.219, *p* < 0.01), fat mass (r = 0.200, *p* < 0.01), and FMI (r = 0.208, *p* < 0.01) and had negatively significant correlation with fat-free mass percentage %FFM (r = −0.219, *p* < 0.01). Among clinical blood parameters, there was a significant positive correlation between Lp(a) levels and total cholesterol (r = 0.210, *p* < 0.01), LDL-cholesterol (r = 0.160, *p* < 0.05), %HbA1c (r = 0.275, *p* < 0.01), and fasting blood glucose (r = 0.2690, *p* < 0.01).

### 3.5. Clinical Predictors of Lipoprotein(a) Level

Based on the correlation analysis we conducted in Table 2, stepwise linear regression was performed to determine the significant predictors of high-risk Lp(a) levels, as shown in Table 3. The stepwise procedure chose a three-variable model for its association with Lp(a): age, total cholesterol (TC), and fat-free mass percentage (%FFM). All three were significantly associated with the outcome; age and total cholesterol (TC) were associated positively, and fat-free mass percentage (%FFM) was associated negatively. Of the three variables, age had the strongest association. For every year increase in age, Lp(a) increased by 0.41 units.

## 4. Discussion

Since lipoprotein(a) levels are highly heritable and vary across races [2], based on our research, our study is the first to explore these aspects in Saudi women. This study identified age as an independent risk factor for women’s elevated Lp(a). While menopause and elevated cholesterol levels were determined as risk factors, the fat-free mass percentage was a protective factor for women’s elevated Lp(a).

Postmenopausal women had higher levels of Lp(a) than premenopausal and perimenopausal women, indicating a relationship between Lp(a) levels and menopause. These findings support previous research findings indicating that the transition to menopause increases the risk of elevated Lp(a) [22]. We were curious about the increased Lp(a) level in our postmenopausal population, so we decided to look into the distribution of high-risk levels in premenopausal and postmenopausal women. We chose 30 mg/dL as there is no universal clinical cutoff value for Lp(a) that distinguishes the increased risk of atherosclerotic cardiovascular development, and several clinical laboratories around the world classify elevated Lp(a) levels as 30 mg/dL and above [23]. When the women were divided into two groups based on their Lp(a) concentration, the proportion of the high risk was highest in postmenopausal women and lowest in premenopausal women, and the amplitude of the distribution doubled in perimenopausal women transitioning to menopause.

Similar to our findings, a large cross-sectional study of Chinese women found that the average Lp(a) level increased with age, and women after menopause had higher Lp(a) levels than women before menopause [24]. The menopause-based differences of high-risk levels of Lp(a) affirm previous observation that Lp(a) may be more atherogenic in postmenopausal compared to premenopausal women.

Premenopausal women have a less proatherogenic lipid profile than men their age; they specifically have higher levels of HDL-C and lower levels of LDL-C and TG [25]. A recent systematic review and meta-analysis were conducted to investigate the effect of menopause on Lp(a) levels, which included 4686 premenopausal and 8274 postmenopausal women, and concluded that transition to menopause might increase Lp(a) concentrations. However, the effect of aging cannot be excluded from the analysis results [22]. The cardioprotective lipid profile is believed to contribute to the female “advantage” during premenopause since men and women have different sex hormones, particularly regarding the availability of estrogen and androgens. Studies examining the impact of specific physiological changes in the hormonal environment in premenopause and postmenopause reveal that these changes are no longer entirely beneficial for women, especially when exogenous sex steroid intake affects plasma lipid concentrations taken into account [25].

Our data analysis revealed that age is positively correlated with Lp(a) levels; this correlation was significant and the highest among other factors. When we compared the correlations among menopausal groups, we noticed that the perimenopausal group had the strongest correlation between age and Lp(a). A plausible explanation for the observed correlation in perimenopausal women could be hormonal changes in the transition to menopause [8,14], in addition to age. Estrogen and its analogs inhibit LPA gene transcription, and hormone replacement treatment was linked to a median 25% drop in Lp(a) in 107 randomized controlled trials in postmenopausal women, which included 33,315 patients [9]. In the Women’s Health Study, Lp(a) at entry was slightly lower among 12,075 women who were active users of hormone replacement treatment (median 9.4 mg/dL) compared to 15,661 women who did not use hormone replacement therapy (11.6 mg/dL) [9,26]. The relationship between age in women and Lp(a) levels was well documented in The Copenhagen General Population Study [7]. Lipoprotein(a) levels were found to increase with age. While the increase in men was consistent across the age range, an additional modest increase in women around 50 was identified, resulting in women having higher lipoprotein(a) levels than men [7].

Interestingly, ROC analysis revealed that age was a significant predictor of elevated Lp(a) in women when the data in our sample were dichotomized at the cutoff age of 50. This reinforces our observation of the highest high-risk level in perimenopausal women, with a mean age of 49.55 ± 1.63. Overall, the role of age in predicting the risk of elevated Lp(a) in women may be more valuable in risk-stratification in clinical practice than relying on menopausal status alone.

Menopause starts with the loss of ovarian hormones and is often associated with changes in adipose tissue distribution, from subcutaneous to more visceral [25]. This study provided new insights into the relationship between Lp(a) concentration and body adiposity. Higher levels of Lp(a) were positively correlated with higher body mass index (BMI) and fat percentage(%BF) and negatively with fat-free percentage(%FFM). Similar to our results, a cross-sectional study on Lebanese children reported that BMI was significantly correlated to Lp(a) levels independent of age [27]. Obesity is typically identified by body mass index (BMI), despite the BMI’s limitation in quantifying body adiposity. When we assess our female cohort by %BF, we found that they score obesity cut-offs by BMI and %BF [28]. Our study found that postmenopausal women had significantly higher BMI, %BF, and FMI than premenopausal women, which can be attributed to the hormonal changes associated with menopause [29]. Menopause generally occurs between 45 and 55 in women and does not happen all at once; it occurs in stages leading up to the postmenopausal phase [29]. Our analysis showed that premenopausal women had higher fat-free mass percentage than postmenopausal women; this is in accordance with several studies that showed that postmenopausal women have lower fat-free mass than premenopausal women [30]. The interplay between the effects of age and menopause on metabolic changes remains an open issue. Aging is associated with a decrease in skeletal muscle loss, which leads to a decrease in basal metabolic rate, considering that skeletal muscles are more than three-fold more active than adipose tissue [31].

Transition to menopause or perimenopause, which usually starts in the mid-forties, is primarily characterized by many hormonal changes mainly caused by a significant decrease in the ovarian follicle numbers followed by a decline in estrogen levels in their fifties [32]. The positive correlation between muscle mass and estrogen has been well illustrated in the literature [32,33]. Nevertheless, Baumgartner et al. [31] noted that estrogen levels were not associated with muscle mass in women aged 65 years and older. Evidence from the literature relating insulin resistance to adiposity and menopause is diverse [32]. In our sample, postmenopausal women have statistically significantly higher glycated hemoglobin (HbA1c) and fasting blood glucose than premenopausal women. It is plausible that the increased cardiometabolic risk in postmenopausal women is driven by insulin resistance, which triggers an increase in inflammation.

According to our analysis, the perimenopausal group had increased total cholesterol and HDL-C values than the postmenopausal and premenopausal groups. LDL-C and triglycerides, however, did not statistically differ between the groups. The association of dyslipidemia with menopause was highlighted in several studies. A meta-analysis of sixty-six studies aimed to compare the lipid profile between premenopausal and postmenopausal women, including 68,394 premenopausal and 46,261 postmenopausal, reported higher total cholesterol, LDL-C, and TG in postmenopausal women than premenopausal. However, no differences were noted in HDL-C levels between the groups. They pointed out that the mean age difference between premenopausal and postmenopausal women was partially attributable to the differences in lipid levels [32]. Due to the concurrent progression of aging and menopause, it can be challenging to distinguish between their independent effects. The remainder of the variance and discrepancies between values could be attributed to either genetic or environmental factors.

Currently, most physicians use lipid-lowering drugs to treat postmenopausal dyslipidemias, focusing more on lifestyle changes that should be crucial in addition to conventional therapy. Controlling two or more modifiable risk factors will provide an extra advantage in preventing atherosclerotic vascular events [25]. Data from the literature show that statin treatment lowers LDL-C levels and cardiovascular morbidity and mortality, regardless of gender. However, there are some differences between men and women in statin metabolism. It has been suggested that women’s glomerular filtration rates are lower than men’s; therefore, women are more vulnerable to the risk of experiencing adverse reactions [25]. Women are more susceptible to myopathy because they have lower muscle mass than men. When compared to men, women have a higher percentage of fat tissue. Lipophilic statins, for instance, have a higher distribution volume and a lower maximum serum concentration [25]. Clinical findings indicate that the risk of non-adherence to statin treatment in women is higher than in men due to statin-induced myopathy. Adjusting the doses based on renal functions and muscle symptoms can optimize statin therapy for women [25].

A diet rich in polyphenols may be beneficial in reducing cardiovascular diseases, according to the antioxidant, anti-inflammatory, antiplatelet, and other pleiotropic actions. As polyphenols improve lipid and inflammatory markers, they reduce the risk of co-morbidities linked to cardiovascular complications. Despite several preclinical and clinical studies on the benefits of polyphenols in reducing cardiovascular risk, convincing proof of polyphenols’ beneficial therapeutic effects in reducing Lp(a) levels is still needed [34].

The Heart and Estrogen/Progestin Replacement Study (HERS) compared hormone replacement therapy with a placebo [35]. Baseline median (quartile 1–quartile 3) Lp(a) levels ranged from 25 (7–55) mg/dL in postmenopausal women with coronary artery disease. With the baseline Lp(a) quartile, the risk for major adverse cardiovascular events (MACE) increased in the placebo group. With hormone replacement treatment, Lp(a) decreased on average by 5 mg/dL, ranging from zero in the quartile with the lowest baseline Lp(a) to about 12 mg/dL in the quartile with the highest baseline. The treatment hazard ratio for hormone replacement therapy was advantageous in patients with a baseline Lp(a) above the median (interaction *p* = 0.03), although there was no overall benefit of hormone replacement therapy on MACE [9,35]. However, estrogen is not recommended to lower Lp(a) levels in postmenopausal women because there is no evidence that HRT generally lowers cardiovascular risk in that cohort. However, similar to aspirin, some available data raise the possibility that hormone replacement therapy might benefit the cardiovascular system in individuals with high levels of Lp(a) [9].

Statins and other lipid-modifying treatments lower cardiovascular risk without significantly lowering Lp(a) levels. On the other hand, lipid-modifying treatments such as niacin reduce Lp(a) levels without significantly affecting cardiovascular risk [9].

Targeted therapeutics for the LPA gene, such as antisense oligonucleotide and small interfering RNA molecules, lower Lp(a) levels. However, their clinical efficacy is being investigated in ongoing clinical trials [9].

Dosing Lp(a) is significant, especially in women and individuals with high or moderate cardiovascular risk. Elevated Lp(a) in men and women is correlated with higher mortality in the high-risk group. However, in women, Lp(a) levels indicate cardiovascular mortality in the moderate-risk group [36]. Further studies are required to understand the nature of Lp(a) and its associated risks in postmenopausal women to improve cardiovascular outcomes.

### Strengths and Limitations

This study has strengths since lipoprotein(a) levels vary significantly between races and ethnicities and, based on our research, this study is the first to investigate the level of Lp(a) in Saudi women and compare it between premenopausal, perimenopausal, and postmenopausal women. The primary outcomes were assessed using objective and validated measurements that are widely used. Using turbidimetric immunoassay, we measured plasma Lp(a) and estimated body fat percentage and fat-free muscle by using bioelectrical impedance analysis. The possible limitations of our study are the relatively small sample size and cross-sectional design, which precludes inference of causality relationship of Lp(a) concentration with age and menopausal status. Moreover, we could not estimate apolipoprotein B100 levels and distribution of apolipoprotein(a) isoforms in the targeted population which might give more explicit evidence of cardiovascular risk.

## 5. Conclusions

We found that lipoprotein(a) levels are associated with age and menopause. Age was a strong predictor of elevated Lp(a) levels in Saudi women. The magnitude of Lp(a) increased significantly after age 50. Menopause and high cholesterol levels were determined as risk factors; the fat-free mass percentage was a protective factor for women’s elevated Lp(a). Lp(a) increased by 0.41 units for every year of age, indicating the strongest correlation.

Postmenopausal women have higher lipoprotein(a) levels than premenopausal and perimenopausal women. High-risk levels of Lp(a) were 57.1% in postmenopausal women and 19.1% in premenopausal women. Similarly, a high body fat percentage is associated with menopausal status. Postmenopausal women have a higher body fat percentage than premenopausal women. Although lipoprotein(a) is mainly determined by genetics, this suggests that elevated lipoprotein(a) level is a relatively more common risk factor in women aged 50 years and above. Further studies with larger samples are recommended to confirm these associations.

## Figures and Tables

**Table 1:** Anthropometrics, clinical and biochemical characteristics by menopausal status.

	All (*n* = 229)	Premenopausal Women (*n* = 68)	Perimenopausal Women (*n* = 63)	Postmenopausal Women (*n* = 98)	
General Characteristics	Mean ± SD	Mean ± SD	Mean ± SD	Mean ± SD	*p* Value
Age (years)	50.61 ± 11.27	37.41 ± 7.22	49.55 ± 1.63	60.45 ± 6.33	<0.001 *
Weight (kg)	77.84 ± 13.74	75.21 ± 10.75	77.67 ± 14.83	79.77 ± 14.66	0.109
Height (cm)	154.21 ± 12.91	157.10 ± 5.59	151.26 ± 22.31	154.11 ± 6.36	0.034 *
Body Composition Indices					
BMI (kg/m^2^)	32.32 ± 5.83	30.64 ± 4.70	32.13 ± 5.89	33.60 ± 6.23	0.005 *
% BF	41.21 ± 6.57	39.01 ± 6.04	40.95 ± 8.01	42.91 ± 5.39	0.001 *
FM (kg)	32.67 ± 9.84	29.69 ± 7.50	32.64 ± 11.02	34.75 ± 10.02	0.005 *
% FFM	58.78 ± 6.57	60.98 ± 6.04	59.04 ± 8.01	57.08 ± 5.39	0.001 *
FFM (kg)	45.17 ± 5.91	45.51 ± 5.73	45.02 ± 5.74	45.02 ± 6.19	0.847
FMI	13.61 ± 4.20	12.10 ± 3.25	13.59 ± 4.59	14.67 ± 4.24	<0.001 *
FFMI	18.72 ± 2.23	18.43 ± 2.02	18.66 ± 1.90	18.96 ± 2.54	0.316
NFFMI	20.23 ± 2.30	19.83 ± 2.09	20.17 ± 1.84	20.55 ± 2.66	0.137
Cardiovascular Risk Factors					
SBP (mmHg)	135.77 ± 19.80	134.72 ± 19.28	136.00 ± 18.73	136.36 ± 20.96	0.867
DBP (mmHg)	81.03 ± 11.49	81.94 ± 11.20	80.30 ± 10.31	80.86 ± 12.45	0.707
Pulse Pressure	54.74 ± 17.12	52.77 ± 17.66	55.69 ± 16.14	55.50 ± 17.41	0.529
MAP	100.03 ± 11.49	100.94 ± 11.20	99.30 ± 10.31	99.86 ± 12.45	0.707
TC (mmol/L)	4.61 ± 0.99	4.35 ± 0.97	5.02 ± 0.79	4.53 ± 1.05	<0.001 *
TG (mmol/L)	1.46 ± 0.84	1.48 ± 0.84	1.50 ± 0.74	1.42 ± 0.91	0.795
LDL-C (mmol/L)	2.72 ± 0.83	2.59 ± 0.83	2.92 ± 0.73	2.69 ± 0.88	0.064
HDL-C (mmol/L)	1.23 ± 0.43	1.27 ± 0.42	1.37 ± 0.42	1.13 ± 0.41	0.002 *
HbA1c %	6.30 ± 1.99	5.42 ± 1.50	6.96 ± 2.41	6.49 ± 1.78	<0.001 *
FBG (mmol/L)	6.54 ± 3.10	5.50 ± 2.57	7.30 ± 3.65	6.77 ± 2.88	0.002 *
Lp(a) (mg/dL)	28.37 ± 11.60	20.98 ± 12.307	29.92 ± 9.53	32.49 ± 9.83	<0.001 *

Note: Data are presented as mean ± SD; *p* < 0.05 is considered significant. Significant p values are marked with asterisk. Abbreviations: BMI: body mass index; %BF: body fat percentage; FM: fat mass %FFM: fat-free mass percentage; FMI: fat mass index; FFMI: fat-free mass index; NFFMI: normalized fat-free mass index; SBP: systolic blood pressure; DBP: diastolic blood pressure; MAP: mean arterial pressure; TC: total cholesterol; TG: triglycerides; LDL-C: low density lipoprotein cholesterol; HDL-C: high density lipoprotein cholesterol; HbA1c: glycated hemoglobin; FBG: fasting blood glucose; Lp(a): lipoprotein(a).

**Table 2:** Spearman’s correlations of lipoprotein(a) levels with demographics, adiposity indices, and lipid profile.

Variables	All (*n* = 229)	Premenopausal Women (*n* = 68)	Perimenopausal Women (*n* = 63)	Postmenopausal Women (*n* = 98)
R value				
Age (years)	0.488 **	0.294 *	0.522 **	0.307 **
BMI	0.137 *	0.056	0.095	0.076
%BF	0.219 **	0.071	0.244	0.104
FM (kg)	0.200 **	0.121	0.125	0.095
%FFM	−0.219 **	−0.071	−0.244	−0.104
FFM (kg)	0.039	0.114	−0.095	0.065
FMI	0.208 **	0.082	0.157	0.104
FFMI	0.055	−0.018	−0.097	0.073
NFFMI	0.057	−0.017	−0.050	0.054
SBP	−0.059	−0.160	−0.093	−0.046
DBP	0.038	−0.080	0.175	0.076
Pulse Pressure	−0.041	−0.091	−0.157	−0.052
MAP	0.038	−0.080	0.175	0.076
TC	0.210 **	0.362 **	−0.047	0.183
TG	−0.009	−0.059	−0.006	0.024
LDL-C	0.160 *	0.285 *	0.112	0.118
HDL-C	−0.030	0.017	0.023	0.062
HbA1c %	0.275 **	0.204	0.235	0.129
FBG	0.269 **	0.097	0.211	0.166
TG/HDL-C	−0.116	−0.135	−0.117	0.094

Note: ** Correlation is significant at the 0.01 level (two-tailed). * Correlation is significant at the 0.05 level (two-tailed). Abbreviations: BMI: body mass index; %BF: body fat percentage; FM: Fat Mass; %FFM: fat-free mass percentage; FMI: fat mass index; FFMI: fat-free mass index; NFFMI: normalized fat-free mass index; SBP: systolic blood pressure; DBP: diastolic blood pressure; MAP: mean arterial pressure; TC: total Cholesterol; TG: triglycerides; LDL-C: low density lipoprotein cholesterol; HDL-C: high density lipoprotein cholesterol; HbA1c: glycated hemoglobin; FBG: fasting blood glucose; Lp(a): lipoprotein(a).

**Table 3:** Stepwise linear regression analysis to determine the significant predictors of high-risk lipoprotein(a) levels.

Predictors		R	R^2^	Unstandardized B	Standardized B	*p* Value	95.0% Confidence Limits
Model 1	Age	0.459	0.211	0.472	0.459	<0.001	0.353–0.592
Model 2	Age	0.489	0.240	0.446	0.434	<0.001	0.327–0.565
	TC			2.007	0.172	0.004	0.659–3.356
Model 3	Age	0.510	0.260	0.411	0.400	<0.001	0.290–0.532
	TC			1.915	0.164	0.005	0.580–3.251
	% FFM			−0.259	−0.147	0.014	−0.464–−0.053

Note: The stepwise procedure chose a three-variable model for its association with Lp(a): age, TC, and %FFM. All three were significantly associated with the outcome; age and TC positively and %FFM negatively. Of the three variables, age had the strongest association. For every year increase in age, Lp(a) increased by 0.41 units. Abbreviations: Lp(a): lipoprotein(a); TC: total cholesterol; %FFM: fat-free mass percentage.

## Data Availability

The data presented in this study are available on reasonable request from the corresponding author.
